# Community-scale slope stability assessment of urbanisation scenarios in North Quito, Ecuador

**DOI:** 10.1007/s10346-025-02608-6

**Published:** 2025-09-24

**Authors:** R. Hen-Jones, C. Zapata, E. Jiménez, E. A. Holcombe, P. J. Vardanega

**Affiliations:** 1 University of Bristol, Bristol, UK; 2 Escuela Politécnica Nacional, Quito, Ecuador; 3 Universidad San Francisco de Quito, Quito, Ecuador; 4 Robertson Geo, Conwy, UK

**Keywords:** Landslide hazard, Rainfall threshold, Physics-based modelling, Urbanisation

## Abstract

Quito is home to nearly three million inhabitants, many of whom live in informal settlements on the steep soil-mantled slopes that surround the city. Within Quito’s multi-hazard environment, the combination of rapid urban expansion onto already landslide-prone slopes—often without adequate urban planning or provision of services—and rainfall associated with the subtropical highland climate, generates a risk to life and infrastructure from landslides. Landslide hazard assessment and mitigation is challenging due to the large area involved, complexity of urban slopes and sparsity of data. In this study, conceptual models of characteristic slope geometries, soil properties and urban features are developed based on three landslide-prone communities in North Quito. These models form the basis for investigating different urbanisation scenarios and the slope stability response to simulated rainfall. They are used to configure the physics-based Combined Hydrology And Stability Model, CHASM, which is run stochastically allowing tens-of-thousands of combinations of slope input factors to be investigated. Analysis of simulation data includes regional sensitivity analysis and the identification of different rainfall-intensity thresholds for triggering landslides. Slope cutting is identified as the primary driver of small-scale urban landslides. Analysis of simulated landslide characteristics allows the trigger mechanisms for different scenarios to be hypothesised and shows a close correlation with the ratio of strata thickness to effective apparent cohesion. These results support revegetation within existing informal communities, and the preservation of existing vegetation and careful design of cut slopes in new settlements.

**Supplementary Information:**

The online version contains supplementary material available at 10.1007/s10346-025-02608-6.

## Introduction

Rainfall-induced landslides pose a significant risk to urban populations in low- and middle-income countries in the tropics where over 80% of fatal landslides occur globally (Froude and Petley 2018). Climate change and rapid urban expansion are predicted to further elevate this risk (Picarelli et al. 2021; Ozturk et al. 2022). In Quito, the capital city of Ecuador, the interactions between the physical environment, high-intensity rainfall events and human development have resulted in landslide disasters throughout its history (Watson et al. 2022; Zapata et al. 2024) – one of the most recent being the La Gasca debris flow which caused 28 fatalities in 2022 (Municipality of Quito 2022).

As well as hydro-meteorological hazards such as high intensity rainfall and landslides, the Quito Metropolitan District (DMQ) is also exposed to seismic and volcanic hazards (Bucheli and Alvear 2018). The city is situated in the Inter-Andean Valley, which runs approximately NNE to SSW for about 400 km and is flanked by the mountains and volcanic complexes of the Cordillera Occidental (to the west) and Cordillera Oriental (east) (Villagómez 2003; O’Rourke and Crespo 1988). Quito’s soils are dominated by the Cangahua Formation which is highly variable due to the complex geomorphological evolution of the region which involved tectonic, volcanic, glacial, weathering and transportation processes. The formation comprises altered volcanic tuffs, ash deposits, pumice, lapilli, palaeosoils, deposits from mudflows and alluvium (Hall and Mothes 1997; Villagómez 2003) with horizons generally of 10 m to 30 m (Iriondo and Kröhling 2007). The cemented structure of Cangahua means that it has a relatively low permeability especially at depth (Creutzberg et al. 1990). However, it shows very little resistance to erosion (Podwojewski and Germain 2005) and can be excavated or eroded to form near-vertical slopes. It is also prone to weathering and disintegrates by slaking (Creutzberg et al. 1990). In the context of urban development, slope cuttings in Cangahua soils may appear stable after initial excavation, but their longer-term stability is likely to be affected by weathering of the cut-slope faces.

The Quito Metropolitan District (DMQ) experienced rapid urbanisation in the second half of the twentieth century with its population increasing sevenfold between 1950 and 2001, largely due to new employment opportunities in the expanding petroleum industry and the development of tropical agriculture (Carrión and Vásconez 2003). The combination of rapid urbanisation and a limited supply of housing has led to ongoing expansion of the city westwards onto the lower slopes of the Pichincha Volcano (Carrión and Vásconez 2003; Riaño 2001; Carrión 1980). Conversely, in the north of Quito, urban expansion has progressed north and eastward from the higher elevations of the anticline formed by the Quito Fault System down into the valleys of Tumbaco, Cumbayá, Los Chillos, Calderón and Pomasqui-San Antonio de Pichincha (Carrión and Vásconez 2003) – meaning that urbanisation here typically starts near the top of slopes and communities expand downslope.

In Quito and similar cities in the tropics, much of the urban development process is informal and unregulated, with communities often classed as “barrios ilegales” meaning that they do not possess official approval. Such communities often lack basic services such as water and sewerage (Carrión and Vásconez 2003). This lack of provision may be sustained by the municipal governments not wanting to encourage residents to occupy high-risk areas (Nguyen 2013). Residents typically turn to makeshift drainage solutions, adding to other slope destabilising factors that often characterise informal hillside settlements including clearing natural vegetation, non-engineered building construction and excavation of slope cuttings, altered natural drainage and blocking of drainage routes with solid waste. Such practices can locally reduce slope stability and lead to frequent landslides even during non-extreme rainfall events (Smyth and Royle 2000; Anderson and Holcombe 2013; Bozzolan et al. 2020; Smith et al. 2020).

Today, up to 60% of Quito’s nearly three million inhabitants live in informal settlements (Municipality of the Metropolitan District of Quito 2017). Within Quito’s multi-hazard environment, the combination of rapid urban expansion onto already landslide-prone slopes, without adequate urban planning or provision of services and a subtropical highland climate generates an increased risk to life and infrastructure. Given the interactions between the physical environment, rainfall and urban expansion in Quito and similar cities, landslide-resilient urban development strategies should preferably be informed by an assessment of: (i) the slope instability factors affecting landslide susceptibility (e.g., geomorphology, lithology, vegetation, hydrological and anthropogenic influences) and (ii) the dynamic slope failure mechanisms – how instability factors interact with each other and with triggering events such as rainfall.

Spatially distributed landslide susceptibility assessments can range in scale from hillsides to cities to regions of over 1000 km^2^. Methods include direct geomorphological mapping; heuristic and index-based weighting or statistical (data-driven) modelling of instability factors in conjunction with landslide inventories; and physically based modelling based on simple representations of slope stability such as the infinite slope method (for a comprehensive review see Corominas et al. 2014, and Reichenbach et al. 2018). Previous studies in Quito have employed direct geomorphological mapping, heuristic, and statistical methods to assess rainfall-triggered landslide susceptibility at a range of scales in the city (e.g., Puente-Sotomayor et al. 2021; Salcedo et al. 2018; Ormaza Nieto 2017; Younes Cárdenas et al. 2016). However, the utility of these types of landslide susceptibility maps can be hindered by an inability to represent localised urban instability factors and by uncertainties or gaps in data such as soil depth, geotechnical properties, or landslide inventories (Maes et al. 2017). Moreover, data-driven methods do not represent future rainfall scenarios, dynamic slope stability mechanisms, or urbanisation activities which can change instability factors locally and over time (UN 2019).

Physically based modelling of individual slope cross-sections can provide insights into dynamic urban landslide causes and potential mitigations measures, but this approach is difficult to scale to city levels. Therefore, in this paper a hybrid modelling approach is applied to address the challenges of spatial scale, data gaps and uncertainties, and process representation in landslide-prone communities in Quito. This approach involves applying the physically based Combined Hydrology And Stability Model (CHASM) – which simulates the effect of dynamic rainfall infiltration and groundwater seepage on the stability of individual slope cross-sections (Wilkinson et al. 2002a, 2002b; Holcombe et al. 2012) – in a stochastic modelling framework (Almeida et al. 2017; Bozzolan et al. 2020). In this way a population of slopes can be simulated which is *statistically representative* of the study area, explicitly accounts for data uncertainties, and allows exploration of future rainfall and urbanisation scenarios (Bozzolan et al. 2020; Ozturk et al. 2022).

The overall aim of this study is to use the stochastic CHASM modelling approach to investigate the drivers of slope instability in typical north Quito communities, and the effect of different urbanisation scenarios – formal (planned and approved) and informal (unplanned). While urban landslides affect peripheral communities around the whole city, this study focusses on north Quito due to the rapid urban expansion here, and differences between urban landslide causal factors in the north and south of the city including rainfall and climate (Vincenti et al. 2012), soils (Guerrón and Tacuri 2012; Hen-Jones et al. 2022; Othman et al. 2023a) and typical slope development patterns (such as from the fault ridges downslope in north Quito). Detailed slope geometry data obtained via drone mapping of the three representative communities is combined with geotechnical information obtained from a recently developed Quito-specific geodatabase (Hen-Jones et al. 2022; Othman et al 2023b). To this end, the following research questions are addressed:

1. What are the drivers of slope instability in communities of north Quito and how are these drivers affected by different urbanisation scenarios?
2. Are differences in urbanisation scenarios reflected in the slope stability response to rainfall?
3. Can landslide size be linked to specific slope instability factors?

**Fig. 1:**
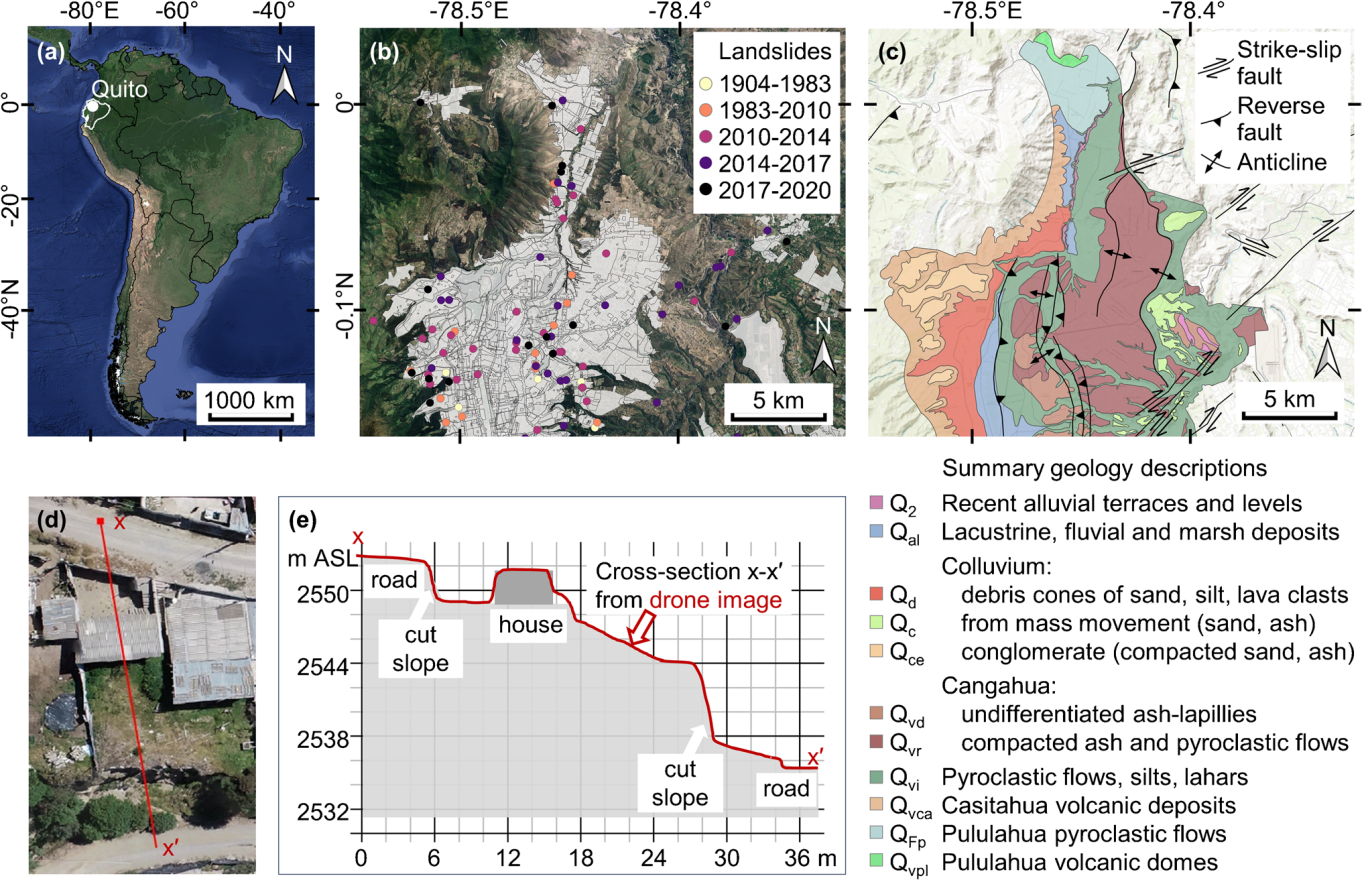
Context for the case study: (**a**) Location of Quito in South America in the Inter-Andean region of Ecuador; (**b**) North Quito urban extent as of 2020, and reported mass movements – landslides, debris flows and subsidence, 1904–2020 (Zapata et al. 2024); (**c**) North Quito topography, Quito Fault System (Alvarado et al. 2014), and geology (Municipality of Quito 2015) with summary descriptions (see Othman et al. 2023a, b, for full descriptions and related geotechnical data); (**d**) drone image of house in an unplanned hillside community in North Quito (copyright Camilo Zapata); (**e**) analysis of cross-section extracted from drone image using Q-GIS profile tool. *Attributions for basemaps*: satellite imagery in (**a**) and (**b**) (Google Satellite imagery © 2024 TerraMetrics) with Ecuador national border at 1:1,000,000 scale in (**a**) from Instituto Geografico Militar Ecuador (https://www.geoportaligm.gob.ec/portal/ last accessed 22 May 2024); Quito urban extent used in (**a**) and (**b**) using data to 2015 from Gobierno Abierto, Secretaría General de Planificación (https://gobiernoabierto.quito.gob.ec/smi/, last accessed 22 May 2024, updated to 2020 by Zapata et al. 2024); topography used in (**c**) from Esri (2012)

The context for this study is illustrated in Fig. 1 which shows the geographic location of Quito (Fig. 1(a)); the topography, urban extent as of 2020, reported landslides and geology of north Quito (Fig. 1(b) and (c)); and a drone image and slope cross-section indicating the typical characteristics of urban development in an informally developed community in north Quito (Fig. 1(d) and (e)).

 .

## Methodology

The workflow for the stochastic physics-based modelling approach adopted here comprises the following steps:

1. **Develop a conceptual model** of hillslope urbanisation in north Quito:
  Identification of communities in north Quito with significant landslide risk.
  Develop a conceptual model of the urbanisation stages, or scenarios, observed in these case study communities upon which to base the **experimental design** of the stochastic simulations.
2. Collate and analyse data related to the case study communities and **generate statistical distributions** for each of the CHASM input parameter values:
  Undertake qualitative and quantitative mapping of the communities to determine slope geometry and urbanisation parameter distributions (also informs conceptual model development in step 1).
  Compile relevant geotechnical information to define the soil input parameter distributions.
3. **Sample the statistical distributions** of the CHASM input parameters using Monte-Carlo analysis to generate realistic combinations of slope geometries, geotechnical properties, urban characteristics and rainfall forcings that together represent the case study communities, slopes and scenarios – creating a library of tens of thousands of ‘virtual’ slopes and rainfall drivers for simulation (i.e., CHASM input files).
4. **Run a CHASM simulation** for each of the virtual slopes in the library (in this case, using the high-performance computing facilities at the University of Bristol), and collate the results for analysis. That is, one set of results per virtual slope. This gives a stochastic simulation library containing tens of thousands of CHASM inputs and outputs that statistically represent the population of slopes and scenarios of interest.
5. **Post-process and analyse** the stochastic simulation library in terms of the sensitivity of slope stability to different input factors and urbanisation scenarios and the slope stability response to simulated rainfall.

The first part of the methodology section develops the conceptual model and experimental design (step 1, above). The physical-basis and numerical scheme of CHASM is then described along with an overview of the different ways the model has previously been used – whether for deterministic or parametric analysis of single slopes, or whether deployed within a stochastic modelling framework to run many thousands of simulations and build libraries of virtual slopes (as is the case in this study). The rest of the methodology section focuses on the CHASM input parameters and their best-fit statistical distributions with respect to the data from the case study communities (step 2), which were sampled to generate the library of CHASM input files for simulation (steps 3 and 4). The data analysis and results section covers the final step of the workflow.

### Development of the conceptual slope model and experimental design

Three typical landslide-prone communities in the north of Quito were used as the basis for this study describing three distinct types of urbanisation scenario:

i. basic informal community with low density housing;
ii. high density unplanned (informal) housing;
iii. low density, planned housing.

The communities selected as being representative of the urban landslide risk context in north Quito were all in locations with steep slopes comprised of cangahua and colluvial soils, at various stages of informal or regularised urbanisation, with socio-economically vulnerable populations, and with a history of slope instability. To develop the conceptual models that formed the basis for stochastic modelling, drone mapping was deployed and high-resolution photographs were captured from which a digital elevation model (DEM) with a pixel size of 10 cm was generated. Natural slope angles and heights, cut slope angles and heights, and house dimensions were extracted from the DEM and the statistical distributions of these measurements were calculated. Qualitative information about vegetation and household drainage systems were determined from the ortho-photomosaics. Additional details of the drone mapping are provided by Hen-Jones et al. (2022).

**Fig. 2:**
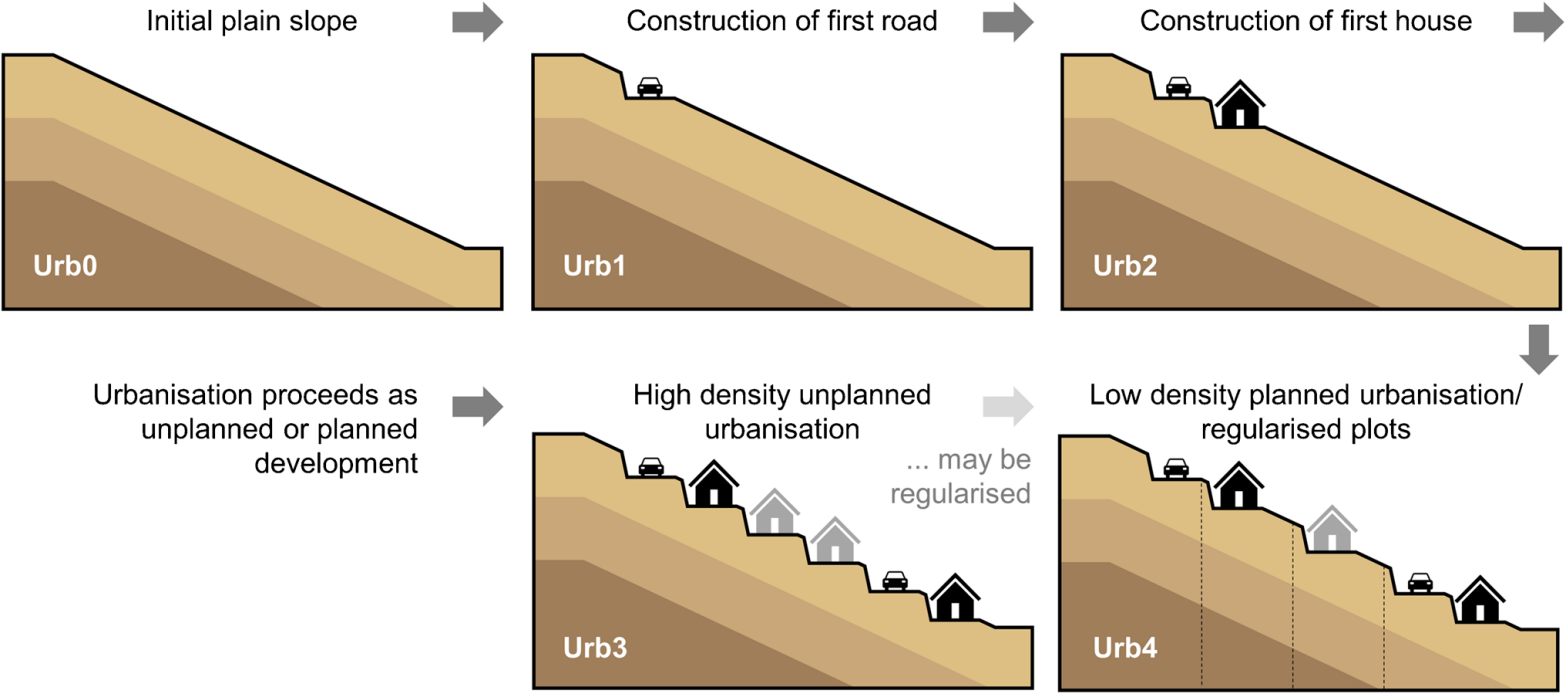
Schematic showing five primary modelled urbanisation scenarios. The presence of features in black is fixed, whilst those in grey are variable

The study sites described above were used as the starting point for the experimental design. The first community was used to model the evolution of the landscape in terms of urbanisation, building up from a plain slope to include the presence of a road and subsequently, minimal housing, thereby allowing the evolution of urbanisation to be modelled in three stages, which serve as the main focus of this study. The second community was used to model high density informal urbanisation; and the third community was used to model low density, planned urbanisation. These degrees of urbanisation translate into five basic scenarios based on the three communities (see Fig. 2):

- Urb0: plain slope;
- Urb1: presence of single road;
- Urb2: presence of minimal housing;
- Urb3: unplanned high density settlement;
- Urb4: planned low density settlement.

The three study sites are in close proximity to one another (all within 500 m of one another), therefore, in terms of materials, identical soil input parameters were implemented across all three communities, with additional surface-level information (slope geometry, housing information) obtained from the drone survey photographs and DEM. These photographs showed both bare ground and grass/shrub type vegetation, therefore for all five geometries, the role of vegetation was investigated by undertaking experiments (i.e. running simulations) with and without the inclusion of shrub-type vegetation. Given the observation of latrines and makeshift sewerage systems in the communities, both the established urbanisation scenarios (Urb3, Urb4) were modelled with household-level leakage to the hillslope, via leaking pipes and tanks, effusing additional water at the surface and below the surface respectively. These investigations translate into twelve individual experiments for each of which 10,000 simulations were generated, as detailed in the Data Analysis and Results section.

### Physics-based modelling and input parameter distributions for stochastic simulations

#### Physics-based modelling of slope hydrology and stability

The Combined Hydrology And Stability Model, CHASM, is a two dimensional physics-based model which computes the evolution of factor of safety (FoS) for a given slope, subject to the dynamic hydrological forcings of rainfall infiltration and groundwater seepage over time. To define a slope cross-section in CHASM a mesh is applied to discretise the slope into cells which are assigned geotechnical parameter values. Initial hydrological conditions are defined by the initial depth of the water table and by the matric suction which is applied at the top of every column of cells. Each timestep (usually between 10 and 60 s) the hydrological component of CHASM uses a finite-difference scheme to solve Richards’ equation (Richards 1931) and Darcy’s Law (Darcy 1856) to calculate the unsaturated and saturated groundwater flow, respectively, and to update the moisture content and negative or positive pore water pressure in each cell. The unsaturated hydraulic conductivities of each cell are updated using soil moisture characteristic, or suction-moisture, curves and the Millington-Quirk procedure (Millington and Quirk 1959). Dynamic hydrological forcings can be imposed during the simulation, such as rainfall of a specified intensity and duration, and leaking water pipes or septic tanks with specified discharge rates (point-water sources).

At the end of each simulation hour the pore water pressures are incorporated into a limit equilibrium analysis of the slope stability. In CHASM Bishop’s circular method of slices (Bishop 1955) is deployed in combination with an automated slip search algorithm which identifies the minimum factor of safety associated with all potential slip surfaces, allowing the factor of safety to be computed for that hour. More details on the numerical scheme and equations used in CHASM can be found in Wilkinson et al. (2002a, 2002b).

CHASM simulations run from time *t* = 0 h for a prescribed number of hours. In this study an initial period of seven days (168 h) was used to allow slope hydrology, as governed by the hydraulic conductivity, saturated moisture content, suction-moisture curve and initial water table, to reach steady state. A design storm was then applied at *t* = 169 h, with the intensity and duration sampled according to the rainfall parameters detailed in Table 1, where the maximum intensity pertains to the approximate maximum observed at a Quito weather station (Escobar-González et al. 2022) – with the duration range set intentionally wide to allow a wide range of synthetic storms to be modelled. Following the end of the design storm, a further four days (96 h) were simulated, which was observed to be sufficient to allow the factor of safety to respond to the design storm and then begin to recover. At the end of each simulation the outputs from CHASM comprise, for each hour: the pore water pressures and moisture contents of each cell used in the slope stability analysis, the minimum factor of safety calculated, the associated slip circle location (centre x,y coordinates and radius), and the weight of slope material within the slip circle (i.e., the landslide weight if *FoS* < 1).

**Table 1:** Slope geometry and soil strata parameters distributions for the north Quito communities, sampled to generate slope input files for the CHASM simulations

**CHASM parameter**	**Symbol (units)**	Statistical distributions* for the urbanisation scenarios shown in Fig. 2				
		**Urb0**	**Urb1**	**Urb2**	**Urb3**	**Urb4**
Natural slope angle	*δ* (°)	LN (3.48, 50.2)	LN (3.48, 50.2)	LN (3.48, 50.2)	U (18.6, 45.4)	N (23.8, 6.94)
Strata thickness of Young Colluvium, YC (upper and lower, I and II), and Old Colluvium, OC	*H*_*YCI*_ (m)	U (5, 8)	U (5, 8)	U (5, 8)	U (5, 8)	U (5,8)
	*H*_*YCII*_ (m)	U (5, 8)	U (5, 8)	U (5, 8)	U (5, 8)	U (5, 8)
	*H*_*OC*_ (m)	U (10, 16)	U (10, 16)	U (10, 16)	U (10, 16)	U (10, 16)
Road cut height	*h*_*road*_ (m)	-	U (5, 10)	U (5, 10)	5	1
House cut height	*h*_*house*_ (m)	-	-	WBL (4.66, 1.57)	LN (1.35, 0.53)	WBL (4.43, 2.16)
Road cut angle	*β*_*road*_ (°)	-	U (70, 80)	U (70, 80)	71.3	U (70, 90)
House cut angle	*β*_*house*_ (°)	-	-	WBL (74.4, 3.28)	84.6	WBL (66.8, 3.42)

*Statistical distribution types: LN (μ_LN_, σ_LN_) = LogNormal (location parameter, scale parameter), N (μ_N_, σ_N_) = Normal (mean, standard deviation), WB (k_WB_, λ_WB_) = Weibull (shape parameter, scale parameter), U (U_min_, U_max_) = Uniform (minimum value, maximum value). Note, where parameters are fixed, this reflects a lack of variability observed from drone mapping results

CHASM has previously been used to assess slope hydrology and stability in various environmental settings and with different modelling approaches depending on data availability, study site and purpose. Examples include a priori deterministic simulations of rainfall scenarios to support a landslide early warning system for a well-instrumented and monitored slope in Germany (Thiebes et al. 2014); parametric analyses of the effects of variations in vegetation and soil properties on slope stability in New Zealand and Hong Kong (Wilkinson et al. 2002a, 2002b); cost–benefit analysis of urban slope drainage in the Eastern Caribbean (Holcombe et al. 2012); probabilistic modelling using random set theory (Rubio et al. 2004); and stochastic modelling to account for data uncertainties in the Eastern Caribbean (Almeida et al. 2017; Bozzolan et al. 2020). This study applies the latter approach to explore practical implications of local slope characteristics and urban development scenarios on slope stability in north Quito, as set out below.

#### Slope geometry and urbanisation parameters

Bozzolan et al. (2020), provide the details of a modification to existing CHASM modelling software allowing urbanisation features to be modelled directly in terms of slope cuts, house loading and household water management features, including functionality to describe gutters, leaking septic tanks and pipes – for full details refer to Bozzolan et al. (2020) and Bozzolan (2021). Road and house cuts are described in terms of the slope height and angle. Where present, leaking pipes effusing at the surface and septic tanks effusing beneath the surface are characterised by the percentage of leakage to the slope. Leakage rates for both are set at a constant rate of 2.3 × 10^–6^ m^3^s^−1^, corresponding to 15% of the estimated total household leakage of low-income housing in St Lucia (Anderson and Holcombe 2013), deemed by the authors to be a reasonable analogy. Houses are described in terms of their unit weight (uniformly sampled between 16 and 48 kNm^−3^, this range set deliberately wide, in order to investigate the significance of house loading), and characterised by downslope pitched roofs, effusing additional water to the slope surface.

The input parameters for building CHASM slope cross-sections are slope height and angle, cut slope dimensions and soil strata depths. For individual slopes these can be specified according to site data. Alternatively, to represent numerous slopes and/or account for uncertainty, a stochastic modelling approach can be adopted in which the statistical distributions of these parameters are sampled. In this study stochastic modelling is used to create tens of thousands of representative slopes for each urbanisation scenario (Urb0 to Urb4, see Fig. 2). In the case of the geometry parameters that define the slope surface, the best fit statistical distributions (for example, lognormal, normal, Weibull or uniform) were determined based on hundreds of measurements of the natural slope and of cut slope heights and angles from the high-resolution DEM, obtained from drone surveys of the case study communities. In the case of the soil strata depths, uniform distributions were used to indicate the likely range of depths. The determination of representative strata thicknesses is described in the section below. Additional information is provided in the supplementary material. Table 1 lists the slope input parameters and their associated statistical distributions for this study.

#### Defining the strata and soil parameters

**Fig. 3:**
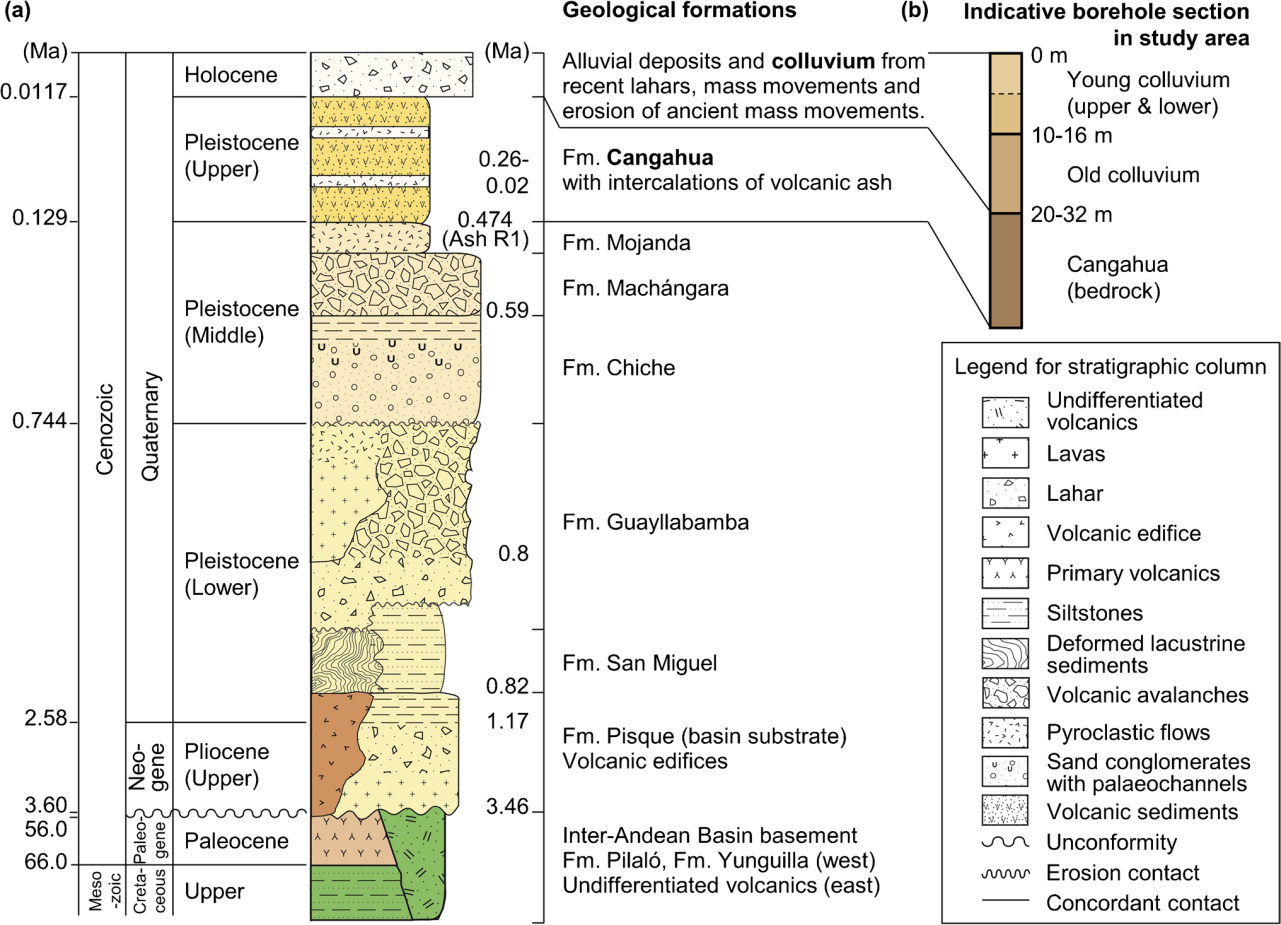
**a** Litho-chronostratigraphic profile of the Quito-Guayllabamba basin based on fieldwork and research by Jiménez 2023 (with reference to earlier studies by Villagomez 2003; Winkler et al. 2005; Vallejo et al. 2019); **b** representative vertical section from case study communities based on site visits, inspection of exposed strata in cut slopes and nearly borehole data

A conceptual model of the lithology and geology in the area of the three study sites was developed in the context of a wider desk-study and review of the geology of Quito, and through several expert-led site visits throughout the study area. Figure 3(a) presents the generalised stratigraphy of the Quito-Guayllabamba basin based on fieldwork and research by Jiménez (2023) and drawing on previous studies by Villagómez (2003), Winkler et al. (2005) and Vallejo et al. (2019) (for recent, detailed lithological maps and sections see Jiménez 2023). The lithology of the slopes of the study area is characterised as consisting of young colluvium (YC) underlain by old colluvium (OC) (derived from erosion and ancient mass movements in Cangahua), underlain by undisturbed Cangahua which represents the bedrock (BR) for slope stability modelling purposes (Fig. 3(b)). These three layers were represented within CHASM with varying depth ranges, as detailed in Table 1, reflecting typical maximum and minimum thicknesses to be stochastically sampled. The young colluvium layer was split into two sublayers (YCI, YCII) to allow soil parameters to be varied with depth within this uppermost stratum.

Key soil input parameters distributions for undertaking modelling of the three North Quito communities were obtained from a preliminary geotechnical database describing the wider Quito area (Table 2), as detailed in Hen-Jones et al. (2022), which provides full details of data types, testing procedures, and statistical analysis (for the most up-to-date version of the Quito geotechnical database see Othman et al. 2023b). For simulations where vegetation was included a shrub-type plant cover is assumed with fixed input parameters: applying an additional surcharge of 0.3 kNm^−2^, a root area ratio of 0.1, root tensile strength of 32 kNm^−2^, and maximum transpiration of 1.5 × 10^–7^ ms^−1^.

**Table 2:** Soil and hydrological parameter distributions for north Quito, sampled to generate slope input files for the CHASM simulations

CHASM parameter	Symbol (units)	Statistical distributions*	
**Soil parameters**		**Soil strata:** Upper Young Colluvium (YCI), Lower Young Colluvium (YCII), Old Colluvium (OC)	**Bedrock stratum (BR)**
Effective apparent cohesion	*c′* (kPa)	LN (3.80, 0.725)	80.0
Effective friction angle	*ϕ′* (°)	N (33.4, 7.02)	60.0
Dry unit weight	*γ*_*d*_ (kNm^3^)	N (14.5, 1.82)	23.0
Saturated moisture content	*θ*_*sat*_ (m^3^m^−3^)	LN (−0.775, 0.129)	As for Old Colluvium, OC, stratum
Residual moisture content	*θ*_*res*_ (m^3^m^−3^)	WBL (0.0742, 3.28)	
Van Genuchten *α*	*α* (m^−1^)	WBL (2.24, 2.72)	
Van Genuchten *n*	*n*	LN (0.395, 0.120)	
Saturated hydraulic conductivity	*K*_*sat*_ (ms^−1^)	LN (−13.8,1.20) for YCI, YCII LN (−14.9, 0.899) for OC	1 × 10^–8^
**Hydrological parameters**			
Initial surface suction	*Ψ*_*init*_ (m)	U (−2.99, −0.50)	
Rainfall intensity	*I* (mm hr^−1^)	U (0, 200)	
Rainfall duration	*D* (hr)	U (1.00,72.0)	
Height of water table	*DWT* (%)	U (42.9, 85.7)	

*Statistical distribution types: LN (μ_LN_, σ_LN_) = LogNormal (location parameter, scale parameter), N (μ_N_, σ_N_) = Normal (mean, standard deviation), WB (k_WB_, λ_WB_) = Weibull (shape parameter, scale parameter), U (U_min_, U_max_) = Uniform (minimum value, maximum value)

## Data Analysis and Results

### CHASM post-processing and analysis

Table 3 summarises the results of the twelve experimental simulation scenarios. For each experiment, the total number of simulations performed was 10,000, however, not all simulations ran successfully, due to the incompatibility of some input parameters when sampled at their extremes within their statistical distributions and combined with other sampled parameter values. Unsuccessful simulations typically comprised 1.0% to 1.7% of the total simulations submitted and were removed from the simulation dataset. Successful simulations, *N*_*succ*_, were then processed as follows, on the basis of the evolution of the factor of safety with time during the simulation.

1. Pre-rainfall stability filter: simulations which failed (i.e. *FoS* < 1) in the 24 h preceding the application of the design storm at *t* = 169 h were considered inherently unstable (i.e. would not exist in reality); the number passing this filter are given by *N*_*filt*_.
2. Post-rainfall stability filter: simulations for which *FoS* dipped below 1 at any point following the application of the design storm were considered to have failed (number given by *N*_*fail*_), those for which this did not occur were considered stable (number given by *N*_*stable*_).

**Table 3:** Summary of CHASM experiments, including calculated slope failure rates (***λ***_***fail***_)

Urbanisation scenario	Experiment	Vegetation	Water leaks	*N*_*succ*_	*N*_*filt*_	*N*_*stable*_	*N*_*fail*_	*λ*_*fail*_ = *N*_*fail*_*/N*_*filt*_* (%)*
Urb0 Plain slope	Urb0_veg	✓		9898	9845	9626	219	2
	Urb0_bare			9950	9845	9256	589	6
Urb1 Road	Urb1_veg	✓		9898	8675	8103	545	6
	Urb1_bare			9898	9558	8338	1220	13
Urb2 Minimal housing	Urb2_veg	✓		9846	7655	7105	525	7
	Urb2_bare			9841	8655	7522	1084	13
Urb3 Unplanned, high density housing	Urb3_veg	✓		9836	9363	8443	920	10
	Urb3_bare			9832	9365	8075	1290	14
	Urb3_veg_leak	✓	✓	9832	9307	8380	927	10
	Urb3_bare_leak		✓	9832	9318	8034	1284	14
Urb4 Planned, low density housing	Urb4_veg_leak	✓	✓	9832	9491	8733	758	8
	Urb4_bare_leak		✓	9844	9496	8286	1210	13

Prior to further analysis, the inputs of failed simulations were then modified such that uniformly sampled input rainfall duration (as described in Table 1) was replaced with ‘effective rainfall duration’, describing the actual rainfall duration after which failure was simulated to have occurred, which could be significantly less than the total rainfall duration used in the simulation.

### Summary of key outputs for each urbanisation scenario

The lowest slope failure rates occur for the plain slope scenario (Urb0), yielding failure rates of 2% (vegetated) and 6% (bare), showing slope cutting to be the most significant factor affecting slope stability when considering all the datasets. The addition of a single road cutting to the slope (Urb1) has the result of approximately tripling the observed failure rate for vegetated slopes and doubling it for bare slopes. With the next urbanisation stage (Urb2), in which the first house and associated cut slope are added along with the road, the effect on the slope failure rate is negligible due to slope stability already having been significantly reduced by the initial road cut. For bare slopes, when comparing the single-house scenario (Urb2) with the unplanned, high density housing scenario (Urb3) there is only a 1% increase in the slope failure rate. However, when vegetation is included in the single-house and unplanned urbanisation scenarios there is a noticeable 4% increase in failure rate with the higher level of urbanisation (Urb3). Simulated leakage from pipes and septic tanks is observed to make almost no difference in terms of failure rate, either with or without the presence of vegetation. It can be seen that the planned urbanisation scenario (Urb4) results in a lower failure rate than the unplanned scenario (Urb3), especially when vegetation is included.

### Uncertainty analysis of CHASM simulation results

Following processing of the simulation results to identify the predicted stable and failed slopes, Global Sensitivity Analysis (GSA) was performed on scenarios Ur0 to Urb2 (all based on the stepwise urbanisation of the same north Quito communities), in order to determine which modelling input parameters dominate the stability response to rainfall for a slope subject to ongoing urbanisation. GSA was carried out using Matlab, implemented via the SAFE Toolbox, as described in Noacco et al. 2019 (see Sarrazin et al. 2017 for presentation of SAFE Toolbox). Specifically, the Regional Sensitivity Analysis (RSA) or Monte Carlo filtering method was applied (see Hornberger and Spear 1981) due to its particular applicability to categorical outputs, where the output in this case is binary (i.e. slope stability or failure). To assess the influence of each stochastically sampled input parameter on the output condition, cumulative distribution functions (cdfs) are derived for each, separated according to output condition; deviation between the two curves is indicative of sensitivity of the model to the specified input parameter, quantified by the Kolmogorov–Smirnov (KS) statistic, constituting the sensitivity index (Chakravati and Roy 1967). Confidence intervals for the sensitivity analysis were calculated as follows: 100 samples (i.e. sets of input parameters defining an individual simulation) are randomly drawn from each dataset, generating 100 corresponding KS statistics for each input parameter. Deviations between the KS statistics for each input parameter sample are used to produce confidence intervals for the sensitivity indices output from the RSA.

**Fig. 4:**
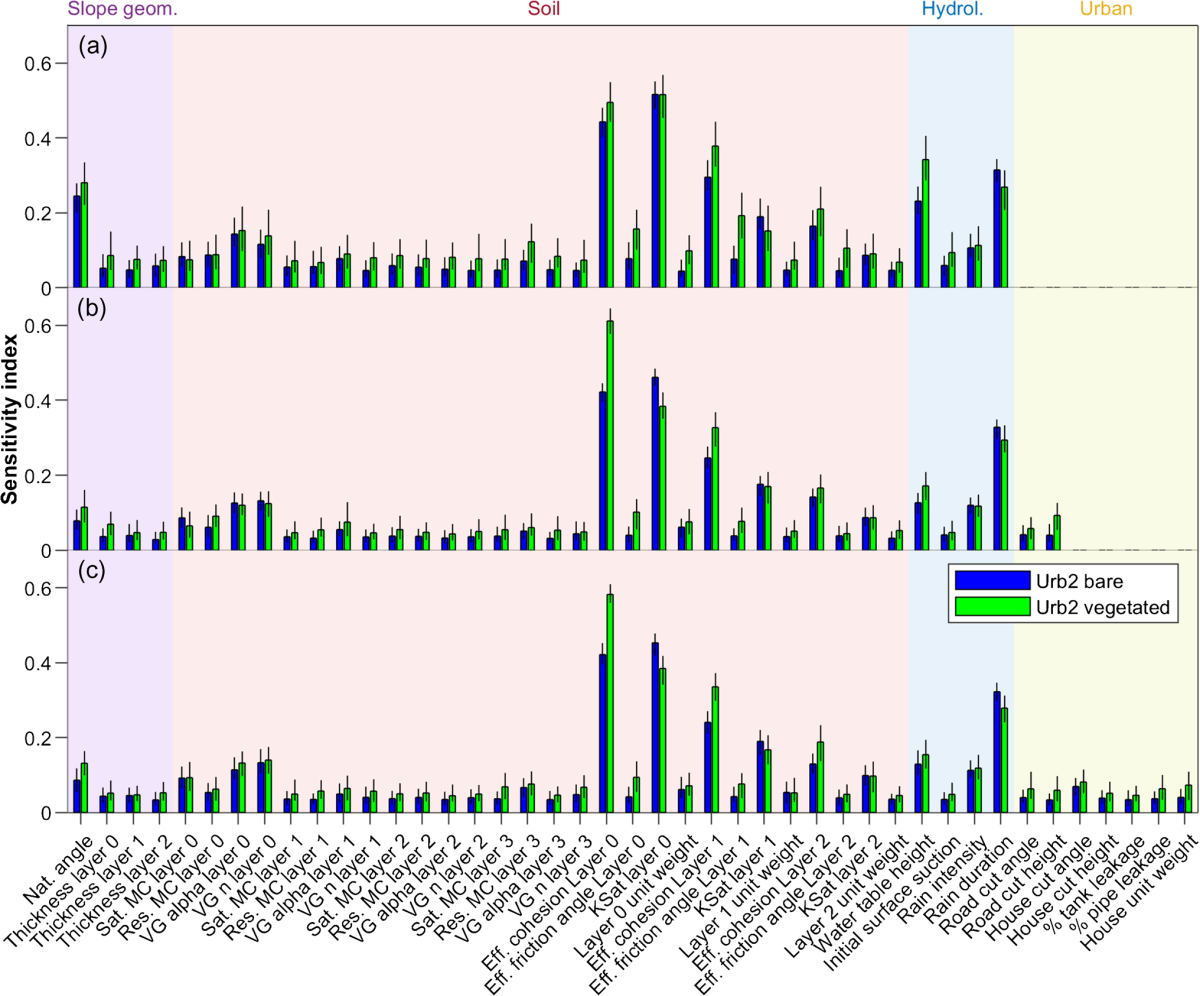
Sensitivity indices according to degree of urbanisation: (**a**) Urb0 – no urbanisation scenario; (**b**) Urb1 – initial urbanisation scenario featuring just one road; **c**) established urbanisation scenario featuring two roads and two houses. For all scenarios, both bare and vegetated scenarios are shown. The variable simulation input parameters are shown on the horizontal axis and are separated into the following categories: geometrical, geotechnical, hydrological and urban. Note, although cut slope heights and angles are effectively geometrical parameters, they have been grouped with other urbanisation parameters

Figure 4 illustrates the results of the Regional Sensitivity Analysis for each of the three urbanisation stages (Urb0, Urb1, Urb2), for both vegetated and bare simulations, grouping geometrical, geotechnical, hydrological and urban parameters together. From the analysis, five key parameters were identified: (i) natural slope angle, (ii) effective apparent cohesion, (iii) saturated hydraulic conductivity (especially of strata layer 0), (iv) water table height and (v) rainfall duration. For plain slopes (Urb0), both natural slope angle and water table height have significant influence on slope stability, however, when the slope is cut into (Urb1, Urb2), they no longer feature strongly as the failure mechanism changes. Across all three scenarios, the principal drivers of instability are the effective apparent cohesion and saturated hydraulic conductivity of the uppermost strata (layer 0, young colluvium I). Considering cut slopes (Urb1, Urb2), effective apparent cohesion dominates when vegetation is present, whilst the saturated hydraulic conductivity dominates for bare slopes, suggesting that vegetation supplements cohesion. For all scenarios, rainfall duration is observed to be key, although the actual intensity of rainfall does not appear to affect slope stability greatly. For all three scenarios, the thicknesses of the slope strata are relatively unimportant. Scenarios Urb1 and Urb2 include urbanisation parameters, none of which feature highly in the sensitivity analysis.

### Calculation of rainfall intensity-duration thresholds

**Fig. 5:**
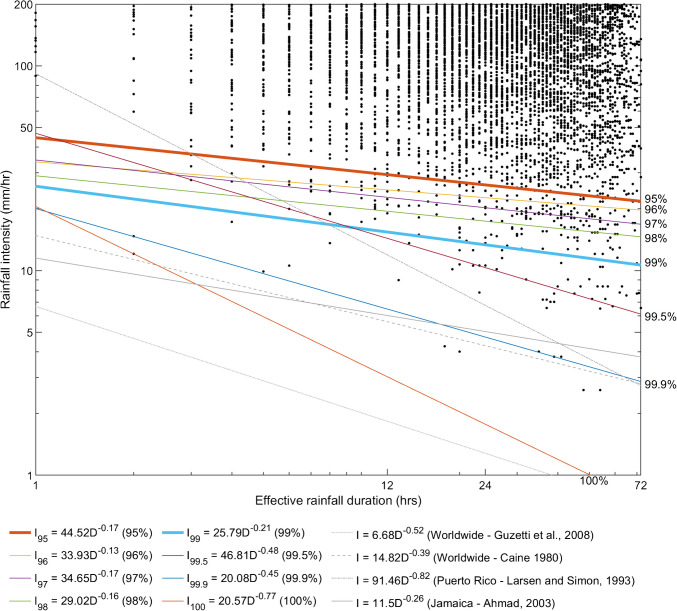
Rainfall Intensity-Effective Duration thresholds for all twelve experimental simulation scenarios. Each point on the graph represents a CHASM simulation in which a slope failure (*FoS* < 1) was predicted as a result of the imposed rainfall. Thresholds bound specified percentages of slope failure data, as indicated. Thresholds constraining 95% and 99% of the data, respectively, are highlighted in bold font. Thresholds from literature are shown in grey

Typically, rainfall intensity-duration thresholds for triggering landslides are deduced from empirical data (e.g. Caine 1980; Larsen and Simon 1993; Guzetti et al. 2008; Ahmad 2003), and describe a critical rainfall threshold below which slope failures are not generally observed. From the aforementioned studies, these thresholds have typically been found to be linear on log–log scales. Due to their empirical nature, traditional thresholds are constructed from a limited number of datapoints and involve the application of a line at the lower bound of datapoints pertaining to failed slopes. In this study, the high volume of simulated data introduces significant scatter to the rainfall intensity-duration plot, necessitating an algorithm to locate a scientifically robust, repeatable threshold. Within Matlab, a multi-objective optimisation algorithm was applied to rainfall intensity-effective duration datapoints for failed simulations, for each of the twelve datasets, in order to define a threshold in terms of intercept, *γ*, and slope, *α*. The two objectives for the optimisation were: (1) maximisation of the number of data points above the threshold; (2) minimisation of the area constrained by the axes and the threshold. By visual inspection of the rainfall intensity-effective duration scatter plot prior to optimisation, a search area was identified in which to constrain the optimisation. Boundaries of *γ* = [−1.699 −0.699] (corresponding to min/max intercepts of 20/200mmhr^−1^) and *α* = [0.1 2] were set. The optimisation algorithm produces a prescribed number (1000) of Pareto-optimised solutions (i.e. slope-intercept combinations), evaluated until the solutions converge. Each of the resultant solutions defines a threshold which constrains a specific amount of the total data. Figure 5 shows rainfall intensity-effective duration thresholds computed by combining all twelve datasets, yielding thresholds constraining varying amounts of data. It can be seen that depending on the prescribed data capture (95, 96, 97, 98, 99, 99.5, 99.9 and 100%), the resultant threshold varies significantly. Thresholds constraining 95% and 99% of the total data are highlighted in bold, allowing for 5% and 1% data scatter, respectively.

**Fig. 6:**
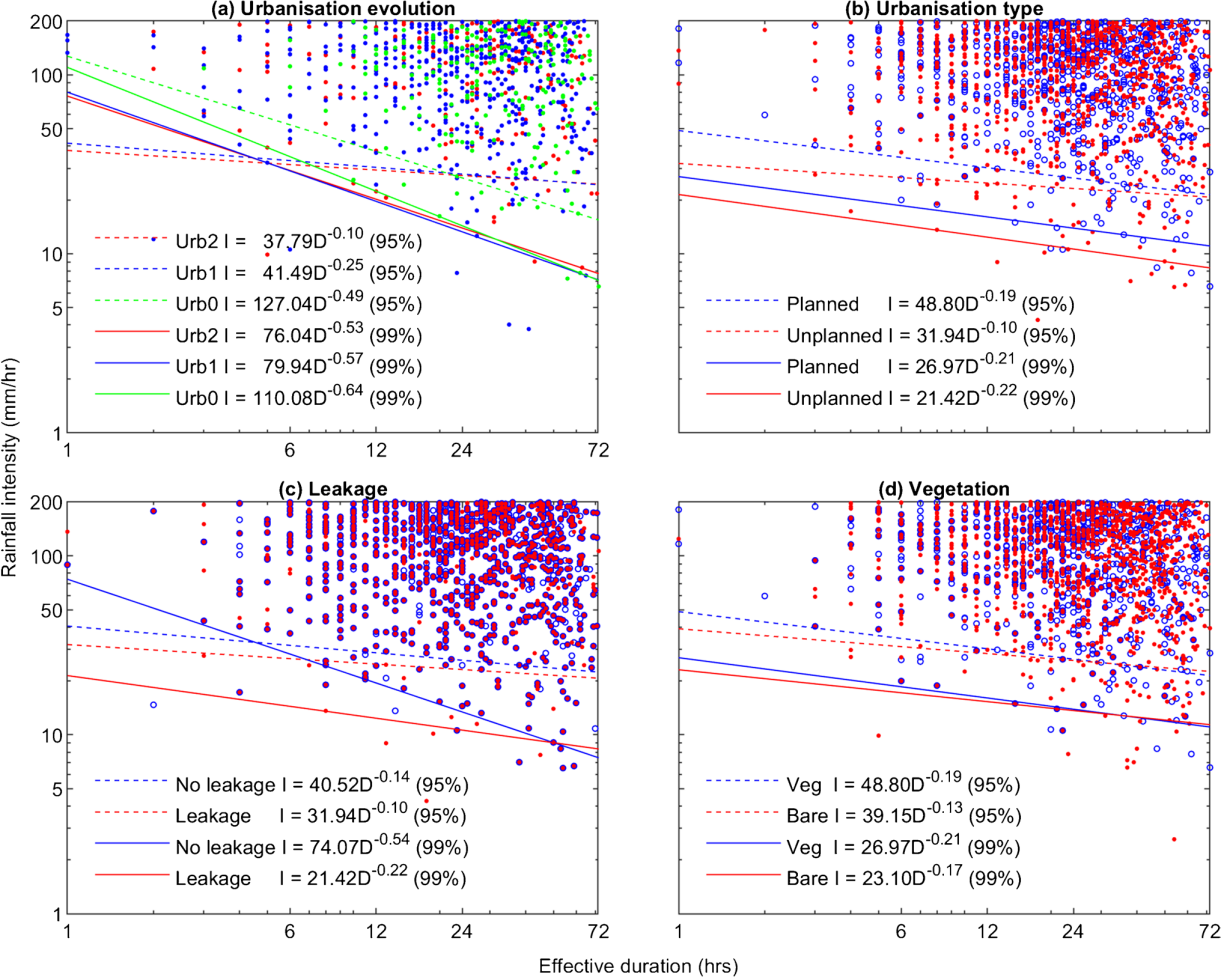
Comparison of landslide-triggering rainfall thresholds showing how different aspects of urbanisation affect slope stability: (a) urbanisation evolution – plain slope (green), single road (blue) and road plus first house (red) scenarios, each with vegetation cover (Urb0_veg, Urb1_veg, Urb2_veg respectively, with corresponding failure rates of 2, 6 and 7%); (b) urbanisation type – planned (blue) versus unplanned (red) scenarios both with vegetation and leaking water simulated (Urb4_veg_leak and Urb3_veg_leak respectively, with corresponding failure rates of 8 and 10%); (c) household water leakage – with (red) vs without (blue), both with vegetation cover for the unplanned housing scenario (Urb3_veg and Urb3_veg_leak respectively, both with failure rates of 10%); (d) vegetation cover – with (blue) vs without (red) for the planned housing scenario (Urb4_veg_leak and Urb4_bare_leak respectively, with corresponding failure rates of 8 and 13%). On these graphs, each point indicates a CHASM simulation in which slope failure (*FoS* < 1) was predicted as a result of the imposed rainfall

In Fig. 6(a), thresholds describing three urbanisation scenarios are presented, and demonstrate a clear offset between non-urbanised slopes (Urb0) and the early urbanisation scenario (Urb1), at both data captures (95%, 99%). Once cuts are present, further cutting and house loading (Urb2) do result in a slight offset of the threshold, but to a much lesser degree. Failure rates between the three scenarios also demonstrate significant increase when cuts are introduced, which then only increase slightly with additional cuts and house loading. The effects of urbanisation type on rainfall threshold are shown in Fig. 6(b), which shows that the planned housing scenario requires more rainfall to fall in order to trigger slope failures than does the unplanned housing scenario – this is reflected in the corresponding failure rates. Separate thresholds pertaining to unplanned housing scenarios, with and without the presence of leaking pipes and tanks, as shown in Fig. 6(c), demonstrate a clear offset between the fitted thresholds, such that slopes with water leakage require less rainfall to fail, however, this is not reflected in the corresponding failure rates. The effects of vegetation on rainfall threshold are shown in Fig. 6(d), which indicates that vegetated slopes to require more rainfall than bare slopes in order to fail, supported by the corresponding failure rates.

### Differences in landslide characteristics 

**Fig. 7:**
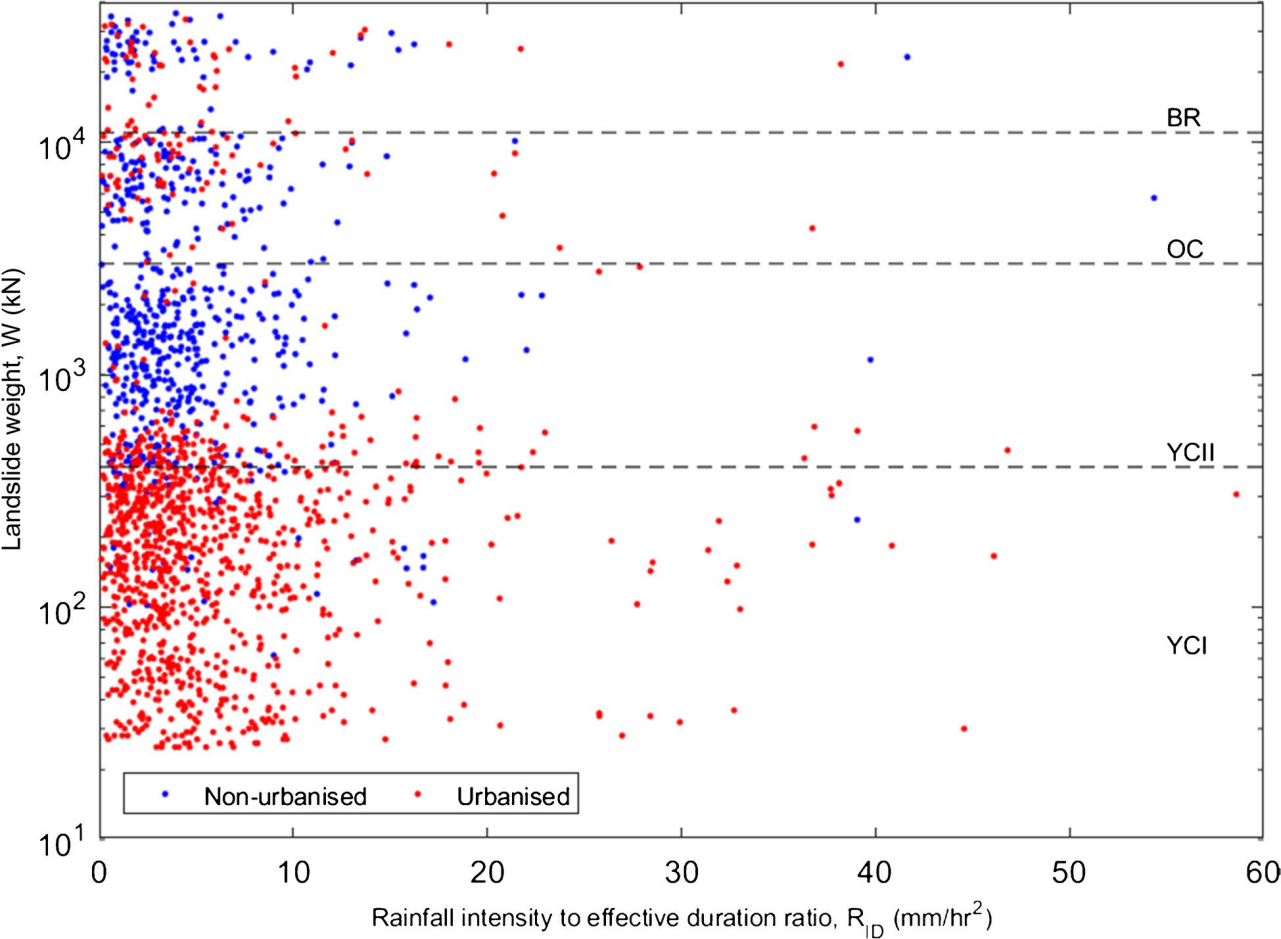
Rainfall intensity-effective duration ratio versus landslide weight for urbanised (red dots) and non-urbanised slopes (blue dots) (simulation scenarios Urb1_bare and Urb0_bare), with corresponding failure rates of 13% and 6%, respectively. Note that landslide weight is on logarithmic axes

**Fig. 8:**
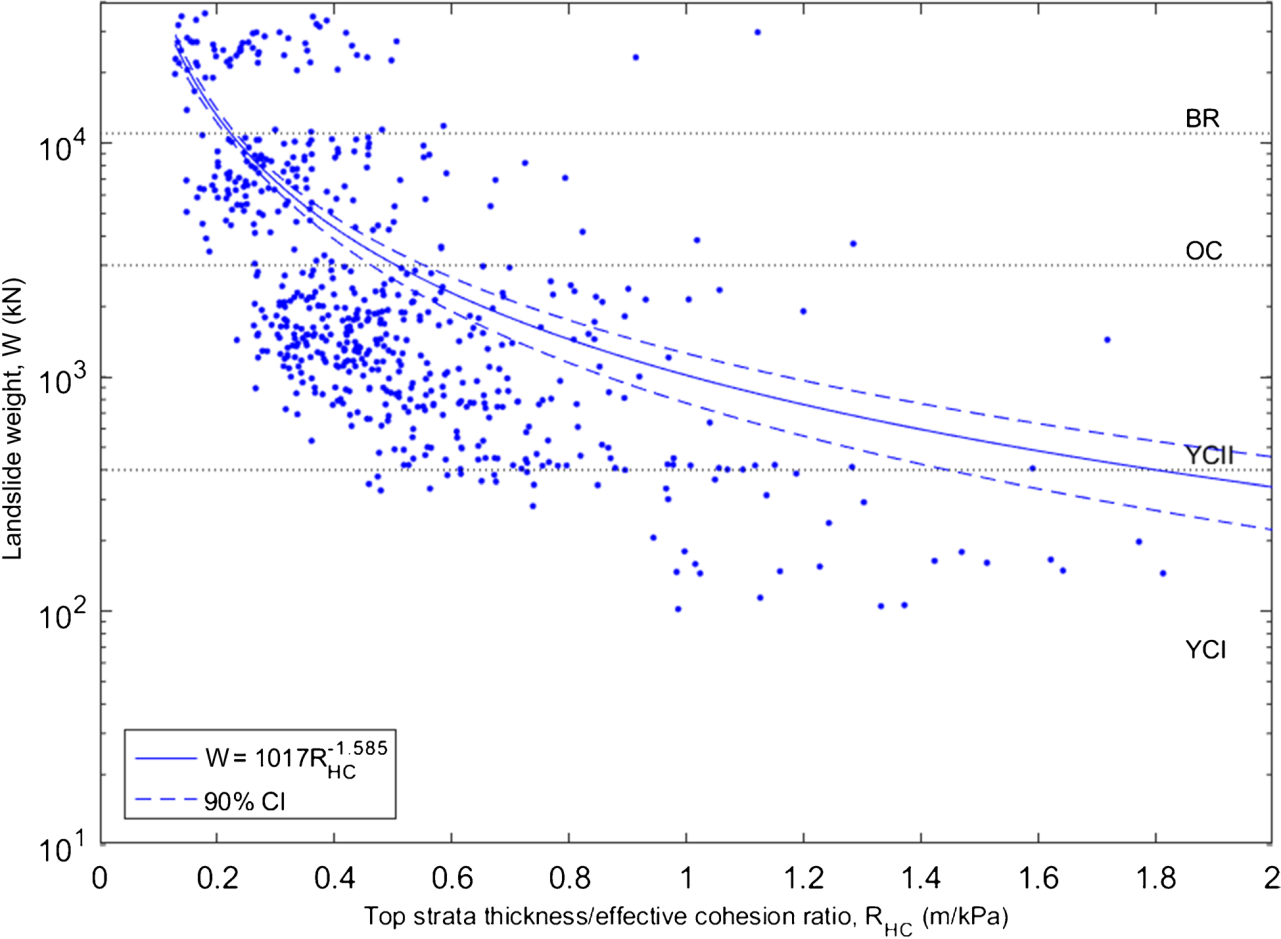
Top strata thickness/effective cohesion ratio versus landslide weight for non-urbanised slopes (Urb0_bare). Simulation dataset has corresponding slope failure rate of 6%. Note that landslide weight is on logarithmic axes. The fitted power trendline was derived from 587 datapoints (after removal of two outliers) and has correlation coefficient r value 0.58 and p value < 0.0001. 90% confidence intervals are shown by dashed lines

At failure, CHASM calculates the weight (*W*) of landslide material, by multiplying the area of the failed segment (intersection of the slope surface and the slip circle) by the discretised unit weights of the soil, assuming a 1 m breadth for the two-dimensional modelled slope cross-section. Analysis of the landslide weight distributions and slip circle locations in terms of simulation input parameters was undertaken to investigate the failure mechanism between urbanised and non-urbanised slopes. The rainfall intensity duration ratio (*R*_*ID*_) has been defined, where low values describe low intensity, long-duration storms, and high values describe high-intensity, short-duration storms. Additionally, the ratio of top strata thickness to effective cohesion (*R*_*HC*_) has been defined, where low values describe thin, strong strata and high values describe thick, weak strata. Figure 7 shows the rainfall intensity to duration ratio plotted against the landslide weight between urbanised and non-urbanised slopes and demonstrates four broad intervals in the landslide weight distribution – these intervals approximately correspond to the strata in which the simulated slopes fail (dashed lines corresponding to landslide weights of 400, 3000 and 11,000 kN respectively). Figure 7 demonstrates the association of small landslides (mostly failing along slip planes within the upper young colluvium layer, YCI) with urbanised slopes, and of large landslides with non-urbanised, plain slopes (mostly failing within the lower young colluvium layer, YCII). These smaller landslides are usually caused by long, low intensity rainfall, but may also be triggered by short, high intensity storms. Larger landslides, in non-urbanised slopes, are rare for short, intense rainfall. In Fig. 8, the top strata thickness to effective cohesion ratio is plotted against the landslide weight. Linear regression was applied to the data, attempting to fit linear, power, exponential and logarithmic models, evaluated by the resulting r and p values. A power trendline has been fitted (Eq. 1) with correlation coefficient (r) value 0.58 (587 data points), and p value < 0.0001.

1 $$W=1017{{R}_{HC}}^{-1.585}$$

## Discussion

### Primary drivers of slope instability on urbanised slopes

Scenarios Urb0 to Urb2 describe the evolution of a natural (unmodified) slope subject to the initial stages of urbanisation (addition of a road and house) and can be considered as the simplest case for assessing the various factors which undermine slope stability. From Table 3, it is observed that the most significant factor which undermines stability is the act of cutting into and oversteepening a section of the slope, as observed in Bozzolan et al. (2020) which investigated the stability of informal housing in the humid tropics. Cutting into the slope has a greater effect on vegetated slopes than on bare slopes, explained by the fact that the process of cutting has the additional effect of removing vegetation, contributing to the destabilisation process. Having been critically steepened by cutting, the addition of further cuts and house loading on the simulated slope cross-section has little further effect on the overall slope failure rate. This is in line with previous studies by Holcombe et al. (2016), who observed a large reduction in factor of safety after the addition of an initial cut, and smaller reductions associated with additional cuts. The jump in failure rate associated with cutting is accompanied by a decrease in landslide size (using weight of failed material as a proxy), as observed from Figs. 7 and 8, indicating a change in typical failure mechanism. Non-urbanised slopes are observed to fail predominantly in the lower young colluvium layer (YCII), where they interact with the water table, which is supported by the results of the sensitivity analysis (Fig. 4) in which saturated hydraulic conductivity, natural slope angle and water table height feature highly. Urbanised slopes fail predominantly in the upper colluvium layer (YCI), independent of the water table, where small landslides occur in cut slopes when preferential failure surfaces are initiated by cutting and localised oversteepening. In such cut slopes, the slope stability is controlled principally by the effective cohesion and saturated hydraulic conductivity of the soil. The rainfall intensity-duration threshold for urbanised slopes found to be lower than that for non-urbanised, plain slopes (Fig. 6(a)), as less rainfall is required for failure. Despite the effect of cutting on slope stability, the urbanisation parameters do not feature highly in the sensitivity analysis, which is likely explained by the relatively narrow ranges of cut heights and angles sampled (Table 2).

After cutting, the removal of vegetation has next the greatest effect on slope stability, evidenced by simulated slope failure rates in Table 3, affecting non-urbanised, plain slopes the most, again explained by urbanised slopes already having some degree of vegetation removal. For the urbanised simulation datasets included in the sensitivity analysis (Urb1, Urb2), the stability response of bare slopes is highly influenced by hydrological parameters (including saturated hydraulic conductivity), in keeping with significant rainfall infiltration into the slope. The stability response of vegetated, urbanised slopes is dominated by effective cohesion, as the interception and evapotranspiration of rainfall by the plant cover mitigates the effects of rainfall and infiltration. Within CHASM, the effective cohesion assigned as a geotechnical property of the slope material and the mechanical effects of vegetation (i.e., the plant roots) are treated separately (Wilkinson et al. 2002a, b), however, vegetation may supplement effective cohesion (Hubble et al. 2013).

The simulated landslides investigated in this study were all triggered by rainfall; therefore, slope stability is naturally related to the input rainfall parameters. Sensitivity analysis demonstrates the importance of rainfall duration, where the infiltration to the slope is controlled by relationship between the saturated hydraulic conductivity and rainfall intensity (Dunne et al. 1991), limiting the sensitivity to the latter. Having established the principal drivers of slope instability, efforts to correlate resultant landslide weight (*W*) with simulation input parameters compared urbanised and non-urbanised slopes with ratio of rainfall intensity to effective duration, *R*_*ID*_ (Fig. 7). It was demonstrated that large-deep landslides were generally associated with low intensity, long duration events, whereas smaller, superficial landslides occurred across a wider range of rainfalls. This observation is supported by Chen et al. (2017), who attribute this observation to larger, deeper landslides requiring an elevated water table for failure, provided by prolonged rainfall. Although top strata thickness does not appear to affect failure rate (from Fig. 4), it does affect the size of landslide; analysis of weight distribution for non-urbanised slopes demonstrates a strong correlation between landslide weight and the ratio of top strata thickness to effective cohesion (R_HC_), formalising the transition from large mass movements in thick, weak layers to small slips in thin, strong strata, with the power relationship given by Eq. 1.

### Implications for slope management

Quito’s rapid urbanisation has seen expansion of the city limits on to steep slopes. Excavation of slope cuts to produce flat surfaces for construction of housing, roads or other infrastructure, is commonplace with urban development onto slopes (Smyth and Royle 2000). Two established North Quito communities were modelled in scenarios Urb3 and Urb4, describing high density, unplanned housing and low density, planned housing, respectively. For the unplanned scenario, observations of identical failure rates with and without additional leakage suggest little benefit from household water management, where a similar lack of sensitivity to leakage was observed in Bozzolan et al. (2020). From Fig. 6(b), a clear offset between landslide-triggering rainfall thresholds simulated for planned and unplanned settlement scenarios indicates that high density, unplanned urbanised slopes are likely to experience slope failures (landslides) for lower magnitude rainfall events than planned settlements. However, the reduction in failure rate associated with vegetation of the free space between cuts is greater than that associated with planning. For both the planned and unplanned housing scenarios, the minimum horizontal separation between cuts was 2 m (parameter *s*_*house*_ as referred to in the supplementary information), which may be too small to allow revegetation slopes in practice; however, where it is practicable, revegetation is certainly indicated to be beneficial for slope stabilisation. Considering the “worst case” of a bare slope with unplanned settlement, the failure rate is 14%; whereas for the “best case” of a planned settlement with vegetation cover, this figure is reduced to 8%.

### Implications of rainfall intensity-duration thresholds

The volume of simulated slope stability data generated in this study required the use of computational methods in order to derive a regional, North Quito rainfall intensity-duration threshold (Fig. 6). Comparison with empirically derived thresholds in the literature shows that these can vary significantly. However, the threshold for Puerto-Rico (Larsen and Simon 1993) shows reasonable agreement with the CHASM simulation-derived dataset and the calculated threshold for North Quito (see also summary presented in Hen-Jones et al. 2024). It can be observed that the prescribed data capture constraint has a significant effect on the resultant fit of the bounding threshold. In order to account for scatter, data constraints of 95% and 99% were applied in Fig. 6(a) to (d), where both values allow trends indicated by varying slope failure rates to be observed. In some cases, however, minimal variation of failure rate was accompanied by a large offset in the computed rainfall threshold, as seen in Fig. 6(c) for the 99% data constraint where little difference in the stability response to rainfall is anticipated. This offset is a function of enforcing a high percentage of data capture to bound the scattered simulation data and demonstrates a pitfall of the automation of the threshold fitting process, despite the scientific robustness of this numerical method versus visual fitting of threshold lines. Where significant scatter is present, lower data capture rules may be appropriate.

## Summary and Conclusions

In this study, different urbanisation scenarios typical of North Quito communities have been successfully modelled using information obtained by drone mapping and demonstrating its merit as a tool in the development of conceptual models, providing information traditionally obtained by walkover surveys and GIS. The most basic type of analysis undertaken in this study is the comparison of slope failure rates between the different urban development simulation scenarios, which has demonstrated that the most important factor in undermining slope stability in the modelled communities is slope cutting, followed by removal of vegetation. Analysis of different urbanisation scenarios supports the importance of revegetation as a policy in landslide management in existing hillside settlements; and that for new settlements, significantly more stable slope profiles result from planned, regulated urbanisation, involving minimal vegetation removal. Synthetic rainfall intensity-duration thresholds for landslide triggering have been fitted to the simulated slope failure data using a computational algorithm, aiding in the quantification of the effects of urbanisation on slope sensitivity to rainfall. However, careful interpretation of these thresholds is required. Analysis of the distribution of landslide weights and locations of slip circles identified different trigger mechanisms and landslide sizes for urbanised and non-urbanised slopes, and strong correlation with the ratio of top strata thickness to effective cohesion.

## Supplementary Information

Below is the link to the electronic supplementary material.

ESM 1 (PDF.316 KB)

## Data Availability

Model input data are specified in the paper. The most recent version of the Quito Geotechnical Database, QUITO/GEO-299 v1.1 (Othman et al. 2023b), is available to download from the University of Bristol Research Data Facility at: < 10.5523/bris.ys9nfsd66knb21h3twx2rfin6 > Datasets for plotting Figs. 4–8 are available upon reasonable request to the corresponding author.
