# Supplementary material for: Community-scale slope stability assessment of urbanisation scenarios in North Quito, Ecuador
Source: Landslides. 2025 Sep 24;23(1):55–71. doi: 10.1007/s10346-025-02608-6 (PMC12764695; doi:10.1007/s10346-025-02608-6)
Supplement: Supplementary file 1 — (PDF.316 KB) [file 10346_2025_2608_MOESM1_ESM.pdf]

Online Supplement

Community-scale slope stability assessment of urbanisation scenarios in North Quito, Ecuador

R. Hen-Jones, C. Zapata, E. Jiménez, E.A. Holcombe & P. J. Vardanega

SUPPLEMENTARY INFORMATION

S1 Modelling north Quito urban slopes and the progression of urbanisation in CHASM

As described in the main body of this paper, three communities in north Quito were modelled to represent three distinct urbanisation scenarios. In the following sections, these typologies are presented in greater detail, in terms of the decisions made and the constraints of the CHASM modelling software. For all of the automatically generated geometries, slopes were modelled as having height h_{slope} = 70m (where one grid spacing within the mesh represents 1m^2). Combined with the modelled natural slope angle, δ (implicitly defining the slope length), the dimensions of the resulting slope geometries allowed the range of urbanisation scenarios to be modelled representing the typical development of north Quito communities starting at the top of slopes and extending downslope, within the constraints of the computational power available (larger slopes are more computationally expensive and take longer to run). An additional 40% of slope height was added to slope base and 20% of slope length was added both upslope and downslope to allow the simulation of the dynamic slope hydrology within CHASM and to avoid any potentially unrealistic hydrological boundary effects from influencing the stability calculation (this was necessary because of the automated generation of tens-of-thousands of slope geometries preventing direct checking and adjustments by the modeller).

Urbanisation scenarios characteristic of north Quito slopes were modelled by addition of an initial road placed at the top of the slope (i.e., nearest to any existing urban areas), and urbanisation features were progressively added going in the downslope direction. The initial road was placed at a horizontal offset s_{road} from the top of slope. The road cut itself is defined explicitly either in terms of its height (h_{road}) or angle (β_{road}), generating a road cut of resulting width w_{road}. House cuts are set a horizontal distance s_{house} from the cut immediately upslope, and as before, are defined explicitly in terms of either their height (h_{house}) or angle (β_{house}, resulting in a house cut of width w_{house}. House loading is implemented in terms of the unit weight of the footprint of the house, which extends across the width of the house cut minus a margin d_{house} at either side. For scenarios involving two roads, the second road is placed a horizontal distance c_{offset} from the first road. This information is detailed in Figure S1 and Table S1. For each of the urbanisation scenarios detailed below, the geometric parameters are given.

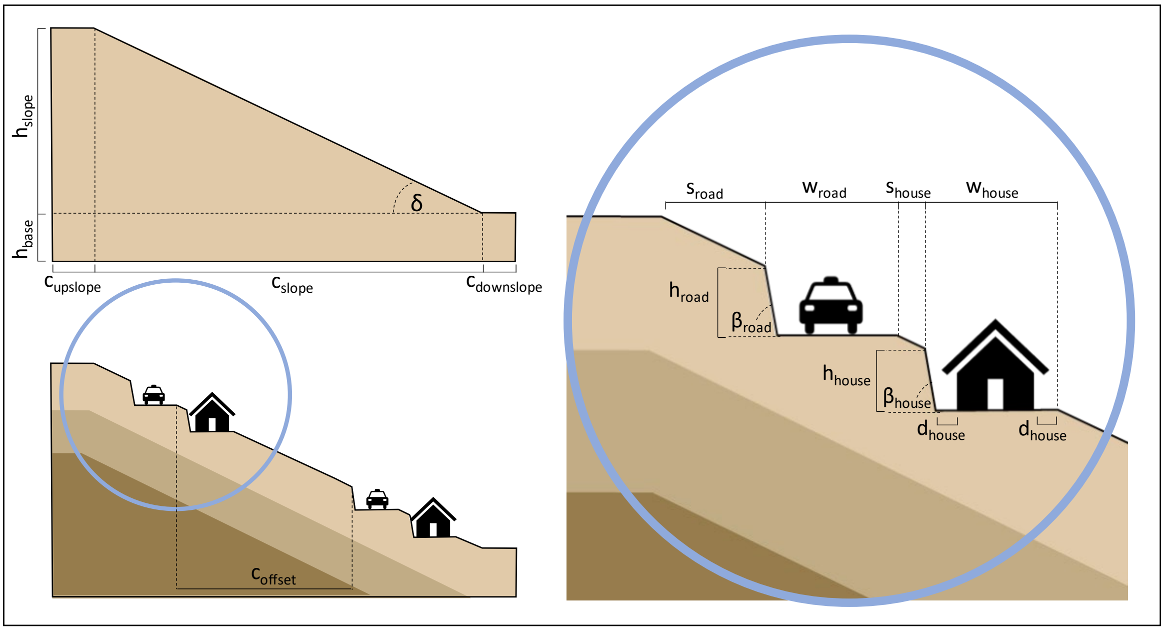

Figure S1. Illustration of geometric and urbanisation modelling parameters.

Table S1. Parameter values common to all scenarios.

**Parameter**	h_{slope}	h_{base}	c_{slope}	$c_{upslope}$ and $c_{downslope}$
**Value**	70	$0.4 \times h_{slope}$	$h_{slope}/tan\delta$	$0.2 \times c_{slope}$
**Units**	*m*	*m*	*m*	*m*

S1.1 Basic community with minimal housing (Urb2)

The most basic initial community development, involving minimal housing, was relatively straightforward to model using the geometric parameter values obtained from drone mapping (see Figure S2 for values). By stochastic variation of the road and house cut parameters (cut angle and height, as detailed in Table 1b), the road and house cut widths are varied indirectly. In order to avoid the generation of geometries with unrealistic house cut widths, additional checks were implemented in order to ensure that house footprints could not exceed max_{house} = 20m (these checks were not necessary for the road cuts as their heights and angles were sampled uniformly, avoiding the generation of unrealistic combinations). Given the concentration of the urbanisation features at the top of the slope, it would have been computationally more efficient to reduce the height of the modelled slopes, however, in order to be able to make direct comparisons with simulations with more downslope features, the decision was made to retain the same slope height across all simulations.

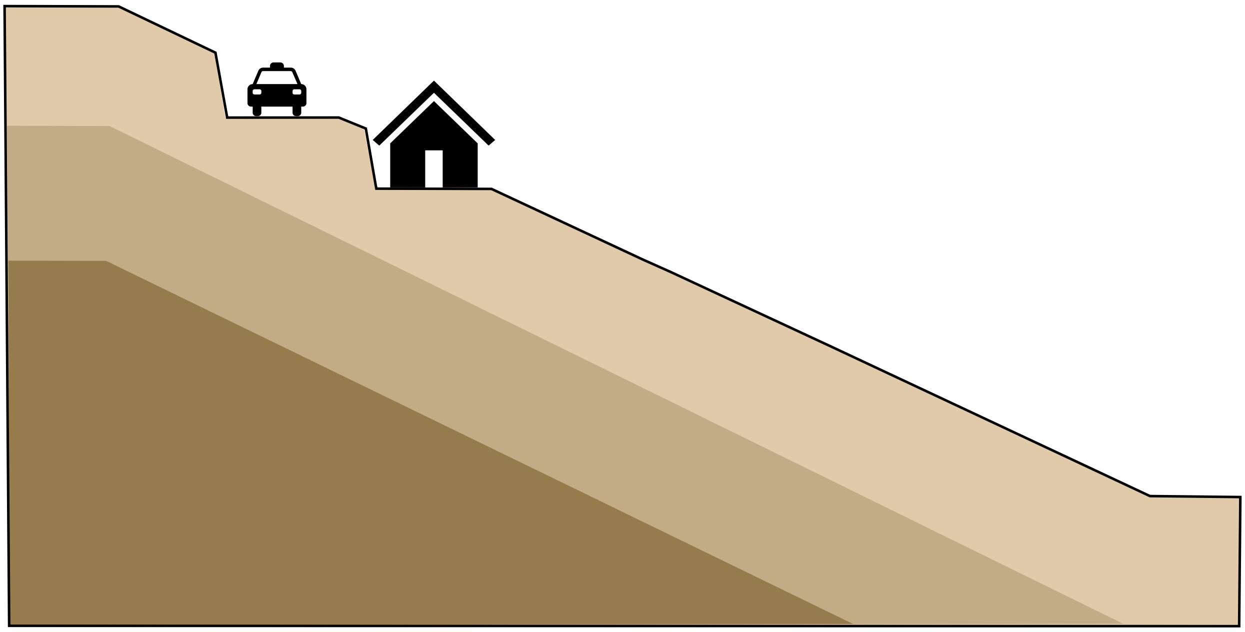

**Parameter**	s_{road}	h_{road}	β_{road}	s_{house}	h_{house}	β_{house}	d_{house}
**Value**	5	U(5,10)	U(70,80)	1	WBL (4.6635, 1.5739)	WBL (74.3592, 3.2844)	1
**Units**	m	m	°	m	m	°	m

Figure S2. Schematic of minimal housing scenario (Urb2). *Statistical distribution types: WB = Weibull, U = Uniform.

S1.2 High density informal/unplanned settlement (Urb3)

The high density informal community modelled in Urb3 involves up to three roads, with mean separation c_{offset} = 35m observed from drone mapping, each road providing access to houses downslope of that road. Due to constraints on the computational power required to model a hillslope sufficiently large to implement three roads and associated housing, the decision was made to model one slope portion only (i.e. housing bound by a road in both upslope and downslope directions). Continuity between slope portions was facilitated by placement of a house downslope of the second road, which was particularly important as slope failures were frequently observed to occur in the cut wall of this downslope-most house.

The number of houses N_{houses} in between the two roads was dictated by a housing density factor, ρ_{house}, prescribing the proportion of the available slope occupied by housing, considering the number of houses possible, N_{house_poss}, in the space between the two roads (c_{offset}), given the total horizontal space required per house cut ($s_{house} + w_{house}$), expressed in equations:

$$N_{house\_poss} = \left\lfloor \frac{c_{offset} - s_{house}}{s_{house} + w_{house}} \right\rfloor \qquad Eq.S1$$

$$N_{house} = \lfloor \rho_{house} \times N_{house\_poss} \rfloor \qquad Eq.S2$$

The housing density was sampled uniformly from 25% to 100%, allowing between one and four houses to be located in the space between the two roads, in keeping with observations made from the drone mapping results. The placement of the houses was dictated by a topdown urbanisation algorithm, which identifies N_{house_poss}

positions where houses could be located, placing the first house in the upslope-most position. The algorithm leaves the next downslope position empty, placing the second house next door but one from the first. When the bottom of the available space is reached, the algorithm returns to the top of the available space and starts to fill in the blank spaces. The algorithm is intended to describe the tendency of residents of informal settlements initially to construct houses which are relatively far from each other, but that as the population of the informal settlement increases, the empty spaces are progressively filled in. Figure S3 demonstrates the implementation of the algorithm for a situation in which the housing density factor ρ_{house} is 100%. From S3, it can be seen that were housing density factor 75%, houses would exist in positions A, B and C; if the housing density factor were 50%, houses would exist in positions A and C only.

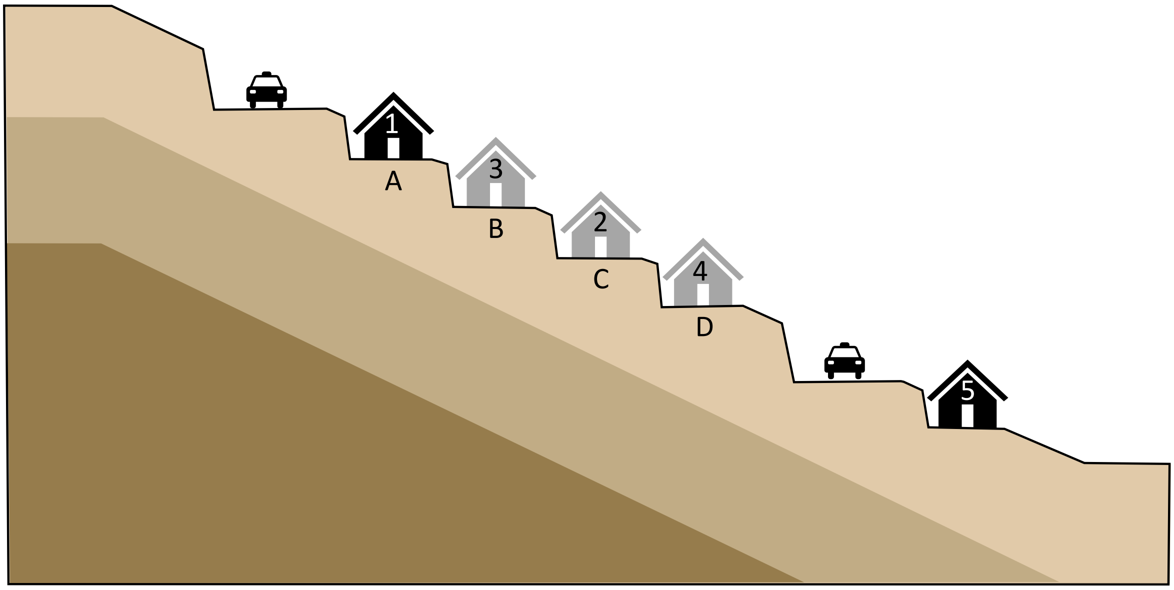

Parameter	s_{road}	h_{road}	β_{road}	s_{house}	h_{house}	β_{house}	d_{house}	c_{offset}
**Value**	5	5	71.27	2	LN(1.3549, 0.5323)	84.6	1	35
**Units**	m	m	°	m	m	°	m	m

Figure S 3. Schematic of high density, informal settlement (Urb3). Grey houses indicate that the presence of the house is variable (dependent on the house density factor); black highlights that the presence and position of the house downslope of the second road are fixed. *Statistical distribution types: LN = LogNormal.

S1.3 Low density, planned settlement (Urb4)

The low density, planned settlement applies a different algorithm by consideration of two house plots within the space between the two roads, describing a planned approach to urbanisation whereby the housing density is limited. In place of the housing density factor, a toggle allows either one or both of the house plots to be constructed on (equal frequency of both options). Where only one plot is urbanised, an additional toggle dictates whether the house is constructed in plot A or plot B (again, equal frequency of both options).

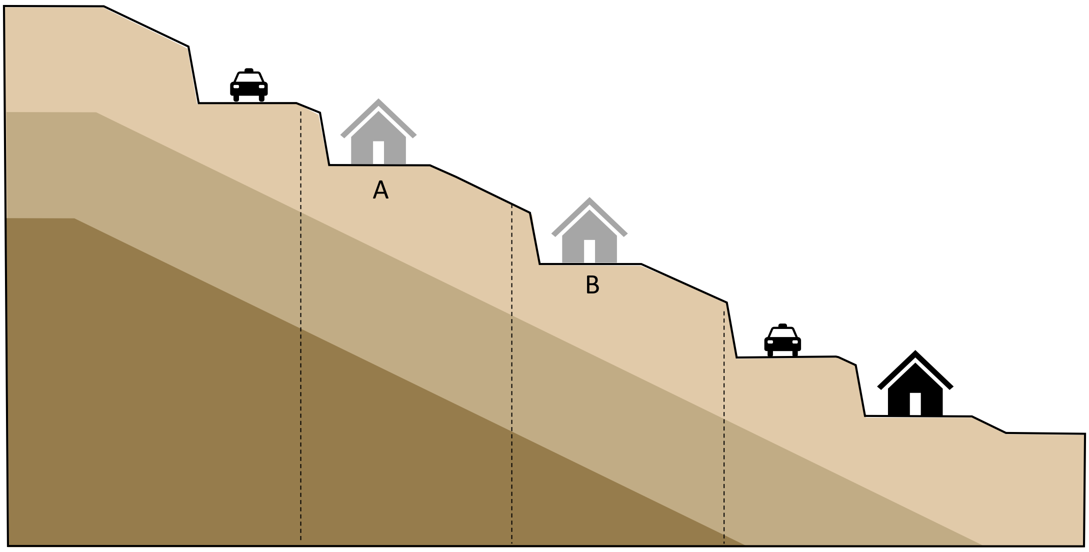

Parameter	s_{road}	h_{road}	β_{road}	s_{house}	h_{house}	β_{house}	d_{house}	c_{offset}
**Value**	5	1	U (70, 90)	2	WBL (4.4339, 2.1629)	WBL (66.7886, 3.4198)	1	35
**Units**	m	m	°	m	m	°	m	m

Figure S 4. Schematic of low density, planned settlement (Urb4). Statistical distribution types: WB = Weibull, U = Uniform.
